# Accurate automatic estimation of total intracranial volume: A nuisance variable with less nuisance

**DOI:** 10.1016/j.neuroimage.2014.09.034

**Published:** 2015-01-01

**Authors:** Ian B. Malone, Kelvin K. Leung, Shona Clegg, Josephine Barnes, Jennifer L. Whitwell, John Ashburner, Nick C. Fox, Gerard R. Ridgway

**Affiliations:** a Dementia Research Centre (DRC), Institute of Neurology, University College London, Queen Square, London WC1N 3BG, UK; b Department of Radiology, Mayo School of Graduate Medical Education, 200 1st St. SW., Rochester, MN 55905, USA; c Wellcome Trust Centre for Neuroimaging, 12 Queen Square, London WC1N 3BG, UK; d FMRIB Centre, Nuffield Department of Clinical Neurosciences, University of Oxford OX3 9DU, UK

**Keywords:** Intracranial volume, Statistical Parametric Mapping, SPM, Freesurfer, Evaluation, Alzheimer's disease, TIV, ICV

## Abstract

Total intracranial volume (TIV/ICV) is an important covariate for volumetric analyses of the brain and brain regions, especially in the study of neurodegenerative diseases, where it can provide a proxy of maximum pre-morbid brain volume. The gold-standard method is manual delineation of brain scans, but this requires careful work by trained operators. We evaluated Statistical Parametric Mapping 12 (SPM12) automated segmentation for TIV measurement in place of manual segmentation and also compared it with SPM8 and FreeSurfer 5.3.0. For T1-weighted MRI acquired from 288 participants in a multi-centre clinical trial in Alzheimer's disease we find a high correlation between SPM12 TIV and manual TIV (R^2^ = 0.940, 95% Confidence Interval (0.924, 0.953)), with a small mean difference (SPM12 40.4 ± 35.4 ml lower than manual, amounting to 2.8% of the overall mean TIV in the study). The correlation with manual measurements (the key aspect when using TIV as a covariate) for SPM12 was significantly higher (p < 0.001) than for either SPM8 (R^2^ = 0.577 CI (0.500, 0.644)) or FreeSurfer (R^2^ = 0.801 CI (0.744, 0.843)). These results suggest that SPM12 TIV estimates are an acceptable substitute for labour-intensive manual estimates even in the challenging context of multiple centres and the presence of neurodegenerative pathology. We also briefly discuss some aspects of the statistical modelling approaches to adjust for TIV.

## Highlights

• 288 T1 MRI from multiple scanners were manually segmented for intracranial volume.
• We compare SPM12 with the current methods of estimating intracranial volume.
• SPM12 shows a very high correlation with manual measures and little bias.
• Newer automated volume measures are more accurate controls for head size variation.

## Graphical abstract

**Figure:**
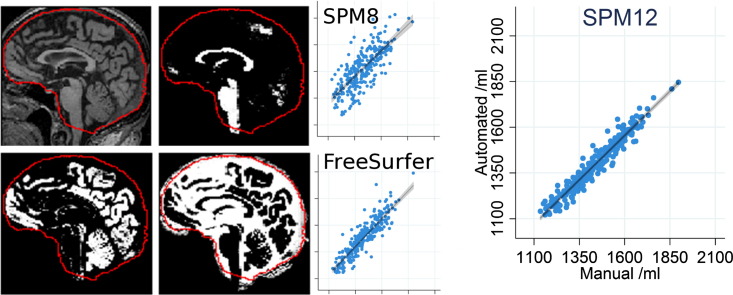

## Introduction

A well-known source of between-subject variability in total and regional brain volume is the variation in head size (Mathalon et al., 1993), often measured by total intra-cranial volume (TIV, equivalently intra-cranial volume: ICV). For example, some of the difference between the sexes in brain volume can be accounted for by differences in TIV (Barnes et al., 2010, Perlaki et al., in press, Whitwell et al., 2001). By modelling otherwise unexplained variability, adjustment for TIV can increase power in studies of overall brain volume (Barnes et al., 2010), total or local grey matter (GM) volumes (Peelle et al., 2012), or individual regions of interest (ROI) (Barnes et al., 2010, Nordenskjöld et al., 2013, Westman et al., 2013). Similarly, TIV can be a confound in the analysis of group differences or covariate correlates if there is an imbalance in head size between groups, an association of TIV with the covariate of interest, or an interaction involving TIV (Ueda et al., 2010). Beyond volumetric analysis, TIV may need to be accounted for in structural connectivity measures (Dennis et al., 2013). In neurodegenerative conditions such as Alzheimer's disease (AD) TIV may be used as a proxy for maximum pre-morbid brain volume, which in turn might relate to cognitive reserve (Perneczky et al., 2010).

TIV may be estimated from volumetric MRI by manual delineation of the cranial vault (for example Whitwell et al., 2001), however this requires trained operators, introduces within- and between-rater variability, and is often impractically labour-intensive when dealing with large numbers of scans. Manual measurement of a covariate is an additional burden to the measurement of the variable of interest (such as ROI delineation). Rapid, reproducible, automatic estimation of TIV based on image registration and/or segmentation has obvious appeal. However, automatically derived estimates could be less useful – or even detrimental – if they are more error-prone than manual estimates or if they introduce systematic biases.

Nordenskjöld et al. (2013) performed an extensive comparison of FreeSurfer 5.1.0 (Dale et al., 1999) and Statistical Parametric Mapping 8 (SPM8, Ashburner and Friston, 2005) on T1-weighted MRI against manual TIV on PD-weighted MRI, with 399 elderly subjects. Despite good correlation, both automated methods were found to have systematic errors compared to manual segmentation, with SPM8 overestimating TIV by 20.86% and FreeSurfer overestimating by 5.87%. These errors were shown to impact the ability to detect differences in hippocampal volume amongst groups. However an improved segmentation was incorporated into SPM8 as the ‘New Segment’ toolbox (Weiskopf et al., 2011 Appendix A); this included additional tissue maps for non-brain soft-tissue, bone and air/background, which help to reduce the amount of non-brain tissue misclassified as the grey matter or CSF. A smaller study (55 subjects) by Ridgway et al. (2011) found that this segmentation produced more accurate TIV results than the previous versions.

In the new release, SPM12, ‘New Segment’ has been made the standard segmentation with further improvements, including changes which may make it more robust to brain volume variation. It is therefore an open question whether the problems identified by Nordenskjöld et al. have been addressed in SPM12; we endeavour to answer this question and to compare SPM12 with the latest version of FreeSurfer.

## Methods

### Data collection

We analysed T1-weighted MPRAGE scans of 288 (m/f 130/158) subjects aged 50–85 collected as part of a “real-world” multi-centre clinical trial (Fox et al., 2005, Gilman et al., 2005). Subjects met the NINCDS-ADRDA criteria for probable Alzheimer's disease (McKhann et al., 1984), mean age was 71.8(7.9) years, MMSE (Folstein et al., 1975) at baseline 20.4(3.3). Scans were coronal volumetric acquisitions lasting ≤ 7.5 min, slice thickness 1.5–1.8 mm adjusted to cover the entire brain, slice FOV 25 × 25 cm, and effective matrix size of 256 × 256 × 124. Acquisition parameters varied over the 17 MRI centres, full details can be found in Fox et al. (2005).

### Manual estimation of TIV

Baseline scans were manually segmented for TIV by four validated operators according to the protocol described in Whitwell et al. (2001), which we summarise here for convenience. The intracranial volume is defined as the “volume within the cranium, including the brain, meninges, and CSF.” Measurements were conducted with the MIDAS software (Freeborough et al., 1997). Whole-brain volumes were first manually delimited using a 3D morphological method (Freeborough et al., 1997).

The T1-weighted volumes are rigidly registered to the Montreal Neurological Institute MNI305 brain average (Evans et al., 2012, Evans et al., 1993). A threshold of 30% of the mean brain signal intensity was used to outline the outer border of the dura as an aid to manual delineation of the outer edge of the intra-cranial volume, and the inferior limit of segmentation is set as the lowest slice in which cerebellar tissue is present. Every 10th axial section was segmented starting from the inferior limit working to the most superior slice with any brain tissue present. Slice areas are linearly interpolated to estimate the TIV for the intervening slices. Intra-rater and inter-rater variabilities were reported to show coefficients of variation (CV) 0.16% (n = 10) and 0.62% (n = 5).

### Automatic estimation of TIV using FreeSurfer

FreeSurfer determines estimated TIV (known there as eTIV or just ICV) using an atlas scaling factor (i.e. the determinant of an affine transformation matrix) derived from registering images to an average template using a full (12-parameter) affine transformation (Buckner et al., 2004; see also http://surfer.nmr.mgh.harvard.edu/fswiki/eTIV). Segmentation is not used. Here, we use FreeSurfer 5.3.0 (the latest stable release as of April 2014), running “recon-all -autorecon1” and obtaining the ICV using “mri_segstats --etiv-only”.

### Automatic estimation of TIV using SPM

There are several methods available to compute TIV using SPM's unified segmentation and spatial normalisation procedure. Methods can be broadly categorised into two main approaches:

1. The spatial normalisation transformation can be used, either inverse-transforming (preserving voxel values rather than volumes) a template-space TIV mask to the individual and determining the volume of the resultant individual-space mask (Keihaninejad et al., 2010) or (equivalently, apart from numerical errors) performing Jacobian integration (Boyes et al., 2006) over a template-space TIV mask.
2. Probabilistic tissue class images can be integrated (i.e. voxels are summed, accounting for the voxel volume) to give tissue volume estimates, with TIV simply being the sum of grey matter, white matter and CSF volumes. A subtlety here is that SPM can provide various sets of tissue class images: native, rigidly-reoriented (and resliced) to standard space, or non-linearly warped to standard space. With volume-preserving transformations^1^ for the latter, all three sets of images should theoretically agree, except for the fact that they can have different fields of view; this can be important, since the amount of e.g. spinal cord contained in the three fields of view can differ, leading to different TIV estimates. The “modulated” non-linearly warped images (with mwc prefixes) should have the most consistent inferior cut-off, which may be the reason for their slightly better performance compared to the “native” subject-space segments in Ridgway et al. (2011).

**Fig. 1:**
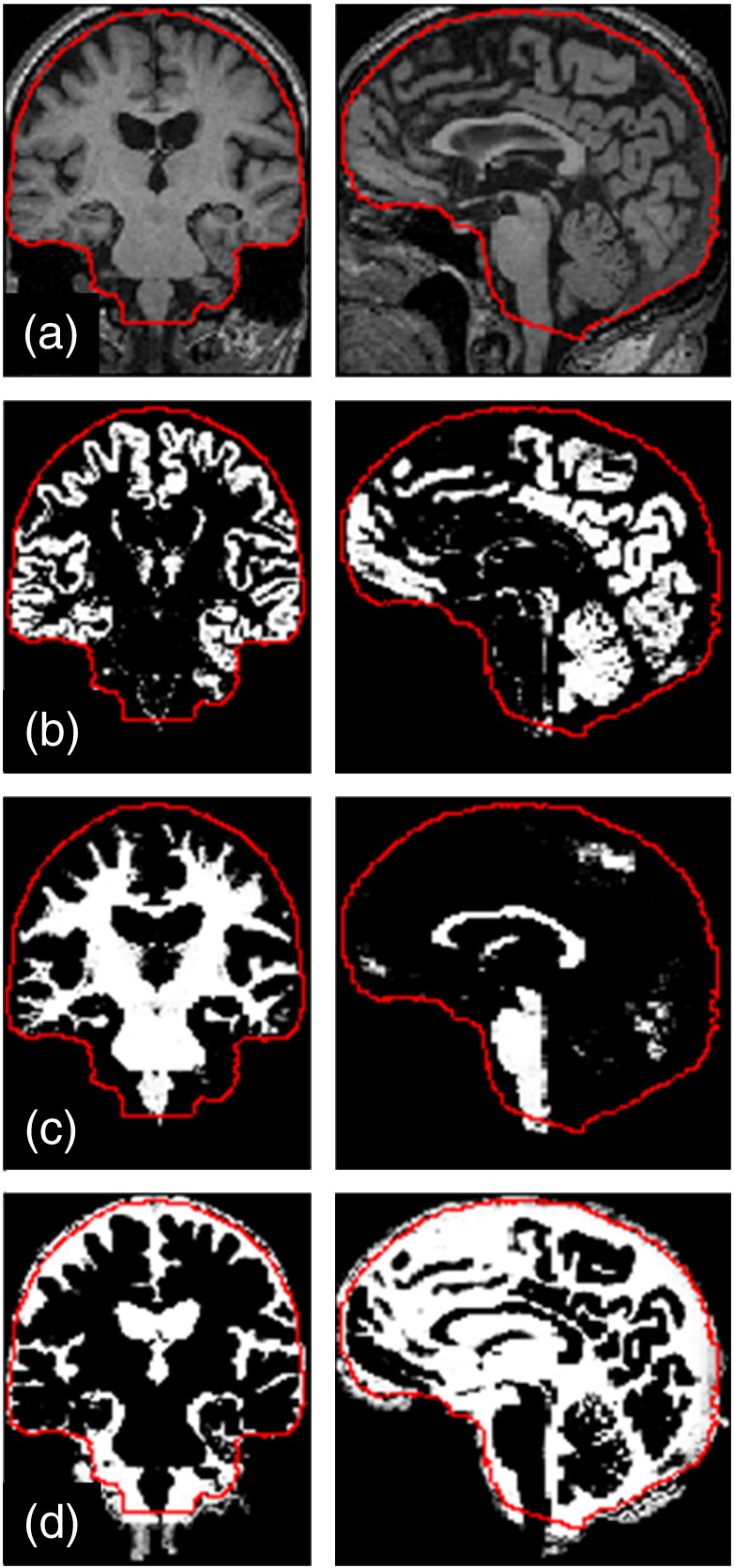
Illustration of SPM12 tissue segmentation results and manually edited intracranial mask: (a) Original T1-weighted MRI [miriad_188],^5^ (b) grey matter, (c) white matter, (d) cerebrospinal fluid; overlaid on each image in red is a contour showing the outline of the intracranial mask after inverse spatial normalisation (i.e. warping from MNI to native space). It can be seen in (d) that the mask excludes some voxels incorrectly segmented as the CSF, and in (c) that the mask achieves a consistent anatomically-defined inferior cut-off, independent of the acquired field-of-view.

It is important to note that the tissue prior probability templates used in SPM are based on averaging multiple automatically segmented images in standard space (for example, SPM12's priors come from segmentations (using New Segment) of images from the IXI data-set, http://www.brain-development.org/ (Heckemann et al., 2003)), so there is no guarantee that the sum of grey matter, white matter and CSF classes will be exactly consistent with accepted definitions of TIV, particularly with regard to the inferior cut-off and the inclusion of blood-filled sinuses. For this reason, we used the SPM12 tissue prior maps (and corresponding average T1-weighted, T2-weighted and proton-density weighted images from the same IXI data) to create a manually-corrected TIV mask consistent with the protocol described above (though segmented at each slice). Fig. 1 shows the TIV mask applied to tissue classes. Supplementary Fig. 1 shows a typical illustration of the non-brain classes, which are almost entirely located outside the ICV.

Although Ridgway et al. (2011) found only very small differences between SPM-based estimates related to approach 1 and approach 2 above, one theoretical advantage of the former is that it yields a contiguous TIV mask, less prone to isolated mis-segmentations far from the intracranial boundaries; a theoretical advantage of the latter approach is that the segmentation can potentially better model finer spatial detail (for example in the slightly more convoluted areas around the temporal lobe and cerebellum) than the regularised (smooth) spatial transformation. In an attempt to combine these advantages, we implemented a “Tissue Volumes” Utility in SPM12, which computes the totals of the modulated warped segmentations within the aforementioned manually-corrected TIV mask. This is available as a built-in SPM utility through the batch editor in the recent beta versions of SPM12.

The unified segmentation algorithm itself in SPM12 is similar to that in SPM8's New Segment, but with recomputed tissue priors (using multi-modal data from IXI, as mentioned above). An additional change is that the global rescaling of tissue priors present in SPM8's default segmentation but not New Segment was reintroduced.^2^ In SPM12, each tissue probability map is rescaled by an additional (non-negative) parameter, and then re-normalised so that the priors sum to one at each point in space. The advantage of this more flexible model of SPM12 is that it allows for global decreases or increases in the amount of each tissue type. This is especially important for dealing with the kinds of atrophy seen in studies of ageing or dementia. The models for old and new segment and SPM12 are further detailed in Appendix A.

SPM12 TIVs were computed using the beta version of SPM12, revision 5647. For comparison, we also use SPM8 (revision 5236), simply summing modulated warped segments without a TIV mask. Finally the Supplementary Table 1 includes results for both the SPM12 Jacobian integration over the TIV mask and volume of the ICV mask transformed to subject-space.

### Statistical analysis

Results were analysed in STATA 12. To assess suitability of automated TIV as a replacement for manual measurements we calculated squared correlation coefficients (R^2^) of automatic with manual measures. As the R^2^ coefficient represents the degree to which variation in each variable is explained by the other, the R^2^ between the two measures indicates the worst-case loss of explanatory power replacing one with the other as a correlate in a linear model. A high R^2^ compared to the gold standard therefore indicates a method that can be used as a proxy for this purpose. Confidence intervals (CI, 95%) on R^2^ coefficients and regression coefficients *β* were estimated using bootstrapping (20,000 samples each test, bias-corrected and accelerated). The same bootstrapping procedure was used to test the paired difference in R^2^ coefficient between the automated TIV methods.

Bland–Altman (B–A) plots (Bland and Altman, 1986) were used to assess the agreement of values from the automated and manual TIV. It is expected that two measures of the same quantity should report the same result, that is: a slope of regression close to 1 (measurement error reduces the measured slope) and differences between measures due only to random error with mean 0 and a standard deviation that is acceptably small. Plotting difference against mean value allows assessment of bias and deviation from parity, using standard t-tests and linear regression. Pitman's test (significance of correlation of difference to mean) was applied to compare variance of the measures.

We do not attempt to compare ICV classification images as two of the methods do not produce them. Our reference, manual segmentation is performed only for every 10th axial section, and the FreeSurfer estimate uses only the atlas scaling factor.

## Results

FreeSurfer failed to register two scans to its atlas correctly, producing TIV estimates > 3000 ml. These two were dropped from the analysis for FreeSurfer, though these subjects were still included for the SPM8 and SPM12 analysis. Mean (SD) manual TIV was 1428.0 (143.5) ml. Table 1 shows correlation and difference for automated methods compared to manual measurements. Direct comparison of R^2^ values using bootstrapping found SPM12 R^2^ significantly higher than FreeSurfer (difference 0.139, CI (0.101 0.194), p < 0.001) and FreeSurfer R^2^ significantly higher than SPM8 (difference 0.224, CI (0.158 0.294), p < 0.001).

**Table 1:** Comparison of automated TIV measures vs manual: squared Pearson's correlation coefficient (R^2^) and slope of regression (*β*), both with 95% confidence intervals, difference to manual ± standard deviation.

	R^2^	β	Difference/ml
SPM12	0.940 (0.924 0.953)	0.971 (0.943 0.999)	− 40.4 ± 35.4 (p < 0.001)
FS 5.3.0	0.801 (0.744 0.843)	1.046 (0.983 1.109)	53.0 ± 74.1 (p < 0.001)
SPM8	0.577 (0.500 0.644)	0.968 (0.878 1.057)	198.3 ± 119.0 (p < 0.001)

**Fig. 2:**
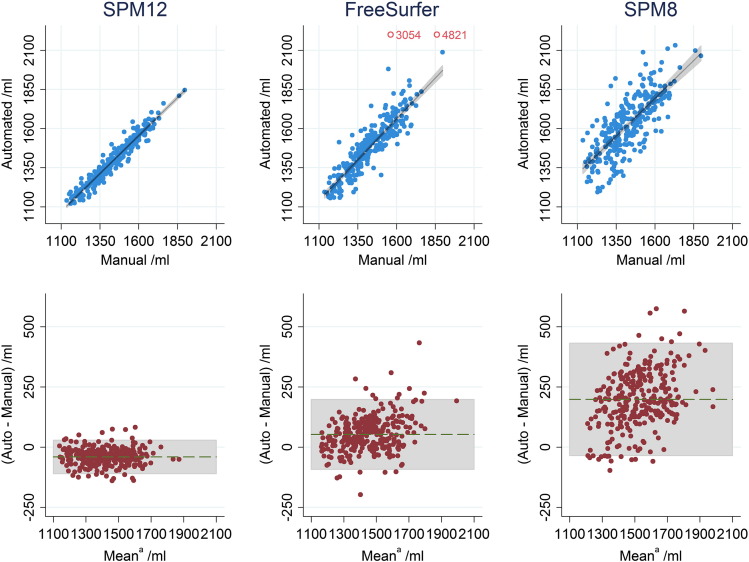
Top: scatter plots of automated TIV vs manual TIV with linear line of best fit (not forced through the origin), and 95% confidence interval for regression line shaded grey. Bottom: B–A plots for automated and manual TIV (^a^automated minus manual plotted against their mean), with 95% limits of agreement shaded grey. Outliers indicated for FreeSurfer by rings are excluded from analysis.

The agreement of the different automated methods with manually delineated TIVs is illustrated in Fig. 2. Pitman's test indicated significant difference of variance compared to manual measure for both FreeSurfer and SPM8 (p < 0.001). SPM12 was the only measure where the results were consistent with the variance being the same (p = 0.95). A significant difference in Pitman's test may be due either to a difference in variance of the two methods being compared or not being bivariate-normally distributed. This cannot be disambiguated without repeated measurements (Bartlett and Frost, 2008, Dunn and Roberts, 1999).

## Discussion

We have compared three automated measures (SPM12, SPM8, FreeSurfer) of TIV with a manual (gold standard) measurement. The high correlation coefficient, narrow limits of agreement and low slope of the B–A plots for SPM12 shown in Fig. 2 suggest that this was the most effective substitute for manual TIV as a covariate in linear models. Results demonstrate a significant improvement over the default SPM8 segmentation and over FreeSurfer. There is a small underestimate in the SPM12 TIV measure compared to manual, which might be due to the blood-filled sinuses being effectively excluded from the tissue segments (i.e. they typically have low probabilities for GM, WM and CSF) even though they are included in the mask. This small bias is of little concern for the use of TIV as a nuisance covariate, since the effect on factors/covariates of interest when adjusting for a nuisance covariate is invariant to affine transformation of that covariate.

Whether the impact of TIV measurement accuracy on a study will be significant will inevitably vary depending on the strength and nature of the underlying relationship, the size of the study, the effect size being measured and the natural variation independent of TIV. In Nordenskjöld et al. (2013) the differences between methods are enough to change the significance of results in a study of 399 subjects. In the Supplementary material we have attempted to simulate the effect TIV measurement error would have on estimated effect size in a simple model of hippocampal volume, this is shown in Supplementary Fig. 2. While there are many factors unaccounted for it remains a good rule of thumb that reducing any measurement errors is a good practice.

### Methods of adjusting for TIV

We have assumed above that adjustment for TIV will be performed by including it as a covariate in a linear model (also known as analysis of covariance, ANCOVA), rather than e.g. dividing regional brain volumes by TIV (known as the proportion method); it is also possible to use a previously fitted regression model (e.g. from a large normative study) to adjust volumes in individuals (known as the residual[isation] method). The relative merits of these methods have been debated in the literature. Arndt et al. (1991) demonstrated problems with the proportion method (reduced joint reliability), and noted that other methods such as regression, “may yield measurements that are more appropriate than ratios”. Mathalon et al. (1993) observed that, “whereas residual scores were uncorrelated with head size (by definition), measures taken as a proportion of head size tended to persist in showing correlations with head size,” which suggests that the former is preferable for adjustment; however, they also note that this property of the latter can be of interest in its own right, in terms of understanding the scaling laws of the brain (see also the discussion of allometry in O'Brien et al. (2006)). Sanfilipo et al. (2004), “found that the residual method generally was less affected by systematic and random errors in ICV and APV [absolute parenchymal volume] values, with the exception of dependent-related APV systematic error”, with the proportion method only being preferable in the latter case. Barnes et al. (2010) regressed logarithmically transformed regional volumes against log(TIV) and found confidence intervals often excluded unity, which further argues against the use of the proportion method; however, the question of whether to log-transform or not arguably remains open.

O'Brien et al. (2011) observe that the ANCOVA and residual approaches have the flexibility to be extended to model a quadratic effect of TIV, allowing for nonlinear relationships between regional volumes and head size. For the case of mass-univariate voxel-wise or vertex-wise analysis, the ANCOVA approach has the advantage that the model can straightforwardly vary over the brain (Barnes et al., 2010, Peelle et al., 2012). Furthermore, the ANCOVA model allows interaction terms to be modelled, e.g. between diagnostic group and TIV (O'Brien et al., 2011, Sanfilipo et al., 2004, Ueda et al., 2010). One could even consider higher order polynomial expansions of TIV – and/or logarithmically or otherwise transformed TIV – interacting with group or other variables. In combination with the above-surveyed advantages, the greater flexibility of the ANCOVA model leads us to recommend it as the first choice to consider. In situations where it is feasible (i.e. no more than a few ROIs) we would also recommend O'Brien et al. (2011) approach of graphically investigating the relationship between regional volume(s) and TIV.

### On the use of “nonlinear-only modulation”

Two popular software packages for voxel-based morphometry, VBM8^3^ and FSL-VBM^4^ recommend a strategy to adjust for head-size by modifying the volume-preserving Jacobian-modulation step such that the affine component is ignored and only the nonlinear volume changes are preserved. This is equivalent to fully preserving the original volume with the usual (affine and nonlinear) Jacobian modulation and subsequently dividing by an estimated TIV obtained from the determinant of the affine transformation. We have not here evaluated the use of the affine determinant from SPM12's unified segmentation to estimate TIV, but there seems no reason to expect SPM12's affine determinant to perform better than FreeSurfer's affine-based estimate.

Since (a) we have shown that SPM12 can provide significantly better estimated TIV than FreeSurfer's affine-based estimate, and (b) we have discussed the limitations of adjustment by division, nonlinear-only modulation may be seen as a convenient but possibly suboptimal procedure.

### Outliers and algorithmic failures

It is often the practice when dealing with automated methods to screen for failures and attempt alternative methods to recover from them (either adjustments to parameters or resorting to another technique). Doing so raises questions of how to define and screen for failures, as well as whether any adjustments should be incorporated as improvements to the method used. On inspecting the range of results we decided to regard as suspicious any estimated TIV greater than 3000 ml and that omitting the two clear failures in the case of FreeSurfer was a fairer comparison of “out-of-the-box” performance. Omission of the corresponding values from the SPM comparisons has little effect on results (Supplementary Table 1). Additionally we attempted to fix the two FreeSurfer failures by suppressing the automated registration checking using the “-notal-check” option for those cases and results for these can be found in Inline Supplementary Table 1. The absence of any notable outliers for SPM12 suggests a good degree of algorithmic robustness.

Only the numerical results were screened for outliers, no routine quality control was applied to the individual images.

### Limitations and further work

We have not directly investigated the effect of atrophy on TIV estimates (cf. Pengas et al., 2009). Our sample population were all probable-AD trial participants with varying degrees of atrophy at baseline (mean manual brain volume:manual TIV ratio 0.69 ± 0.05, min 0.56 max 0.81); the high agreement with manual measures shown by SPM12 is apparently not affected by this. Both Nordenskjöld et al. (2013) and Ridgway et al. (2011) find only small variability over time for manual TIV measures. However, further longitudinal evaluation (as in Pengas et al. (2009)) of the SPM12 method could provide greater reassurance that tissue loss does not change TIV estimates.

Although a large number of different sites and scanners were included here, without obvious detriment to the results, all scanners were 1.5 T, so we cannot claim to have demonstrated robustness to different field strengths (cf. Keihaninejad et al., 2010).

Whilst we have shown very good agreement between SPM12 and manually-measured TIV, we have not directly evaluated the effect of replacing manual with SPM12 values in investigations of other factors such as hippocampal volume as in Nordenskjöld et al. (2013), though the simulations in Supplementary Fig. 2 shed some light on this.

It would also be of interest to evaluate SPM12's performance on data other than T1-weighted MRI; for example, Pengas et al. (2009) and Nordenskjöld et al. (2013) favour proton-density (PD) weighted imaging, whilst Vuong et al. (2013) shows the advantages of T2-weighted MRI. Multi-spectral segmentation of quantitative multi-parametric maps Weiskopf et al. (2013) would be expected to yield even better results, since the combination of PD with other contrasts (including R2*, related to T2) should enhance the distinctions between brain tissue and blood-filled sinuses, and between the CSF and bone/air. It is plausible that automatic methods, perhaps with further refinements, could actually yield more accurate measurements than the current manual gold standard, especially with the latter's use of only every 10th slice, however, demonstrating this would be challenging, requiring somewhat indirect evaluation.

## Conclusions

We have shown that TIV estimated using SPM12 correlates very strongly with manually-traced TIV, providing superior performance to TIV estimates from SPM8 or FreeSurfer. For regional and mass-univariate volumetric studies, we recommend the use of TIV as a covariate in a linear model, which enables the consideration of nonlinearities (i.e. with TIV and TIV-squared) and/or interactions between TIV and other terms.

## Appendix A Changes to the unified segmentation algorithm

### A.A Old and new segment in SPM8

The “old segment” (OS) algorithm of SPM8 is essentially the same as the default tissue segmentation procedure in SPM5 (Ashburner and Friston, 2005). The “new segment” (NS) algorithm of SPM8 is based on the same principles, although some changes were made to the original algorithm; these changes are briefly described in Appendix A of Weiskopf et al. (2011).

Both implementations produce probabilistic segmentations of images into *C* tissue classes, where each class is defined by a tissue probability map that is warped into alignment with the image. The *C* = 4 tissue probability maps of OS (grey matter, white matter, CSF and other) were extended in NS to include tissue priors for bone, non-brain soft-tissue and air (*C* = 6), which resulted in greater robustness in terms of registering the tissue priors to the image. The other main extensions were an increased flexibility of the registration part of the model (by using more parameters), and the ability to do multi-spectral segmentation by simultaneously modelling multiple images of the same subject (eg. T2-weighted and PD-weighted).

When modelling a single image, both versions estimated a correction for intensity nonuniformity, parameterised by coefficients ***β***. In what follows, we denote the nonuniformity correction at voxel *i* by *p*_*i*_(*β*). Intensity distributions for each tissue class are modelled by a mixture of Gaussians, such that the means and variances are adjusted to account for the correction field.

Each of the *K*_*c*_ Gaussians of each tissue class is described by its mixing proportion (*γ*_*ck*_, such that *γ*_*ck*_ ≥ 0 and ∑_k=1_^K_C_^ *γ*_*ck*_ = 1), a mean (*μ*_*ck*_) and a variance (*σ*_*ck*_^2^). With this model, the probability of observing a voxel of intensity *y*_*i*_ in the image is given by:

(A.1) $$p\left(y_{i}|\gamma_{c},\mu_{c},\sigma_{c}^{2},\beta_{c}\right)=\sum_{k=1}^{K_{c}}\frac{\gamma_{c_{k}}}{\sqrt{2\pi \sigma_{c_{k}}^{2}/\rho_{i}{\left(\beta \right)}^{2}}}\,\exp\left(\frac{-{\left(y_{i}-\mu_{c_{k}}/\rho_{i}\left(\beta \right)\right)}^{2}}{2\sigma_{c_{k}}^{2}/\rho_{i}{\left(\beta \right)}^{2}}\right)\text{.}$$

In both cases, warping the tissue maps into alignment involves estimating a vector of coefficients (*α*) that parameterise displacement fields. The *i*th voxel of the warped version of the *c*th tissue prior is denoted by *b*_*ic*_(*α*). For NS, the likelihood model that is maximised (with suitable regularisation) is simply:

(A.2) $$\varepsilon_{n}=\sum_{i=1}^{I}\,\log\left(\sum_{c=1}^{C}b_{ic}\left(\alpha \right)p\left(y_{i}|\gamma_{c},\mu_{c},\sigma_{c}^{2},\beta_{c}\right)\right)\text{.}$$

For OS, there is effectively an additional set of *C* scaling parameters that are estimated, *η*, such that *η*_*c*_ ≥ 0 and ∑_c=1_^C^ *η*_*c*_ = 1. This model accounts for situations where there may be globally more or less of some particular tissue type.

(A.3) $$\varepsilon_{o}=\sum_{i=1}^{I}\,\log\left(\sum_{c=1}^{C}\frac{\eta_{c}b_{ic}\left(\alpha \right)}{\sum \eta_{d}b_{\mathrm{id}}\left(\alpha \right)}p\left(y_{i}|\gamma_{c},\mu_{c},\sigma_{c}^{2},\beta_{c}\right)\right)\text{.}$$

### A.B Segmentation in SPM12

The absence of these scaling parameters has impacted the behaviour of NS in SPM8. This issue has been pointed out numerous times on the SPM mailing list, and is the likely cause of some of the findings in Callaert et al. (2014) as well as Peelle et al. (2012). The issue has now been resolved in the beta version of SPM12, as the segmentation algorithm of SPM12 is essentially NS, but with the *η* parameters included again. Other changes from NS to SPM12 include the new tissue probability maps (described earlier) and a change to the regularisation of the spatial transformation model. The single-parameter bending energy regularisation mentioned in Appendix A of Weiskopf et al. (2011) has been extended to a more sophisticated regularisation model in SPM12, which now has five penalty terms: absolute displacement, membrane energy, bending energy, linear elasticity and divergence; though the bending energy has the largest weight by default. SPM12 also reintroduces a version of the morphological clean-up procedure from OS, and includes a Markov Random Field (MRF) based clean-up that was introduced in later versions of NS in SPM8, but was not documented in Weiskopf et al. (2011). For the present work, the key changes are the tissue priors and the scaling parameters.

## Footnotes

5 http://www.ucl.ac.uk/drc/research/miriad.

2 This was partly motivated by results reported in Peelle et al. (2012), and partly on the basis of unpublished experiments we performed using the MIRIAD data (Malone et al., 2013).

3 http://dbm.neuro.uni-jena.de/vbm8/, see also http://dbm.neuro.uni-jena.de/vbm/segmentation/modulation/.

4 http://fsl.fmrib.ox.ac.uk/fsl/fslwiki/FSLVBM/UserGuide.

## Footnotes

1 Often referred to as “Jacobian (determinant) modulation”, which was used in SPM5, although SPM8's New Segment and SPM12 actually preserve volume with a push-forward transformation procedure akin to the RAVENS maps (Davatzikos et al., 2001).
