# Supplementary material for: Accurate automatic estimation of total intracranial volume: A nuisance variable with less nuisance
Source: Neuroimage. 2015 Jan 1;104:366–72. doi: 10.1016/j.neuroimage.2014.09.034 (PMC4265726; doi:10.1016/j.neuroimage.2014.09.034)
Supplement: Supplementary file 1 — Supplementary material. [file mmc1.doc]

|  | R^2^ | *β* | Difference^c^ /ml |
| --- | --- | --- | --- |
| n=288 |  |  |  |
| SPM12^a^ | 0.940 (0.924 0.953)^b^ | 0.971 (0.943 0.999) | -40.39±35.35 |
| SPM8^a^ | 0.577 (0.500 0.644) | 0.968 (0.878 1.057) | 198.34±118.98 |
| FS 5.3.0 | 0.460 ( 0.359 0.779) | 1.289 (1.050 2.093)^d^ | 68.14±204.69 |
| FS 5.3.0 with outlier fixes | 0.805 (0.750 0.845) | 1.044 (0.983 1.106) | 53.25±74.05 |
| SPM12 Jacobian | 0.918 (0.898 0.934) | 0.975 (0.942 1.008) | -9.16±42.03 |
| SPM12 ICV mask transformed | 0.913 (0.883 0.931) | 0.977 (0.944 1.009) | -9.41±43.44 |
| N=286 (outliers dropped) |  |  |  |
| SPM12 | 0.941 (0.925 0.953) | 0.967 (0.939 0.995) | -40.79±34.72 |
| SPM8 | 0.566 (0.489 0.632) | 0.963 (0.871 1.056) | 198.05±119.34 |
| FS 5.3.0^a^ | 0.801 (0.744 0.843) | 1.046 (0.983 1.109) | 53.05±74.07 |

Supplementary Table 1, comparison of automated TIV measures vs manual: squared Pearson's correlation coefficient (R^2^) and slope of regression (*β*), both with 95% confidence intervals, difference to manual±standard deviation. Outliers were defined as TIV greater than 3000ml, and occurred only for FS 5.3.0; we attempted to fix these by suppressing the automated registration checking using the “-notal-check” option for those cases. In addition to the methods in the main paper we also tested Jacobian-integration with SPM12 using only the ICV mask (no tissue classes) and transformation of the ICV mask back to subject-space.

^a^Also in Table 1.

^b^SPM12 tissue volumes TIV R^2^ with manual significantly higher than for all other methods listed.

^c^All t-tests versus manual TIV p<0.0001, except SPM12 Jacobian and mask, p<0.001.

^d^Extreme outliers strongly affect confidence interval estimation and sensitivity to seed, non-BCA ci (0.89 1.69)


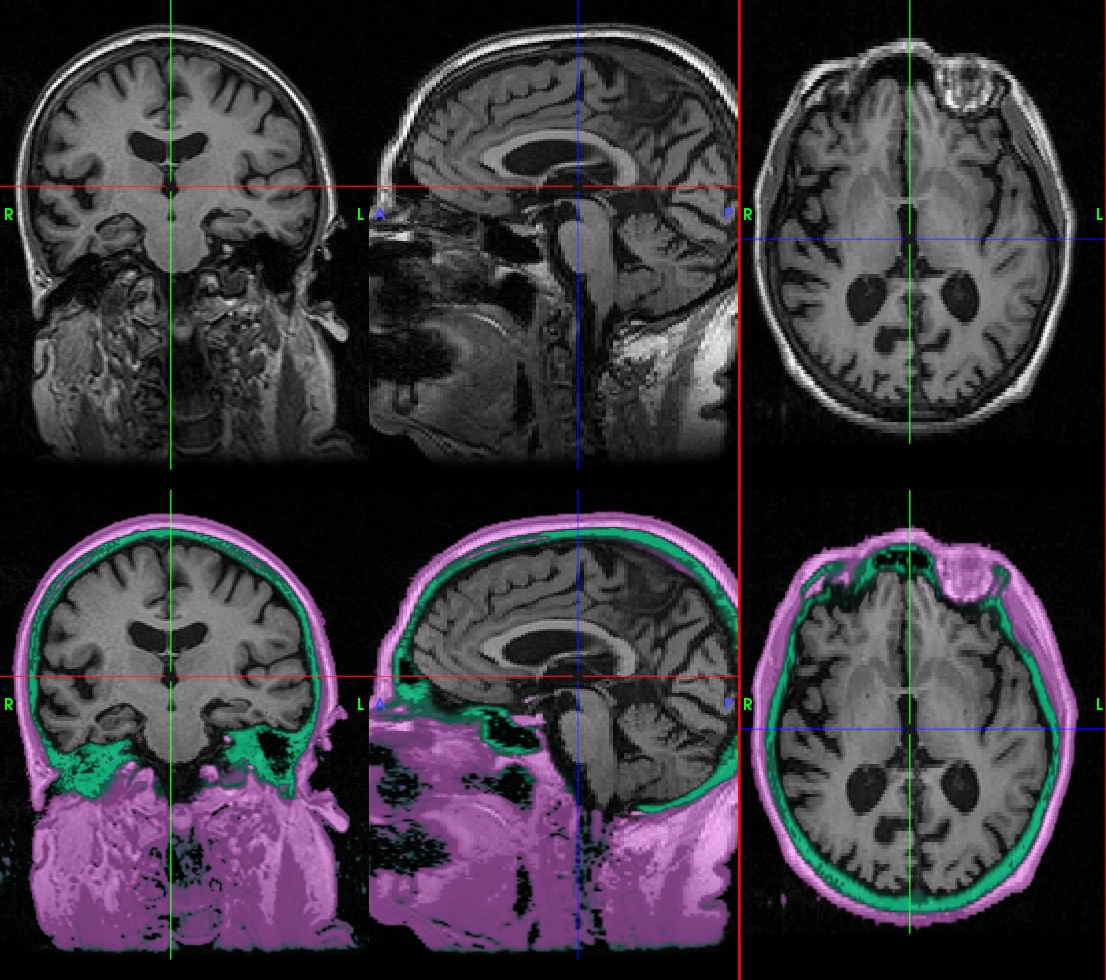
Supplementary Figure 1, non-brain tissue classes, bone (green) and soft tissue (purple). Within the ICV very little soft tissue occurs and mainly represents dura.


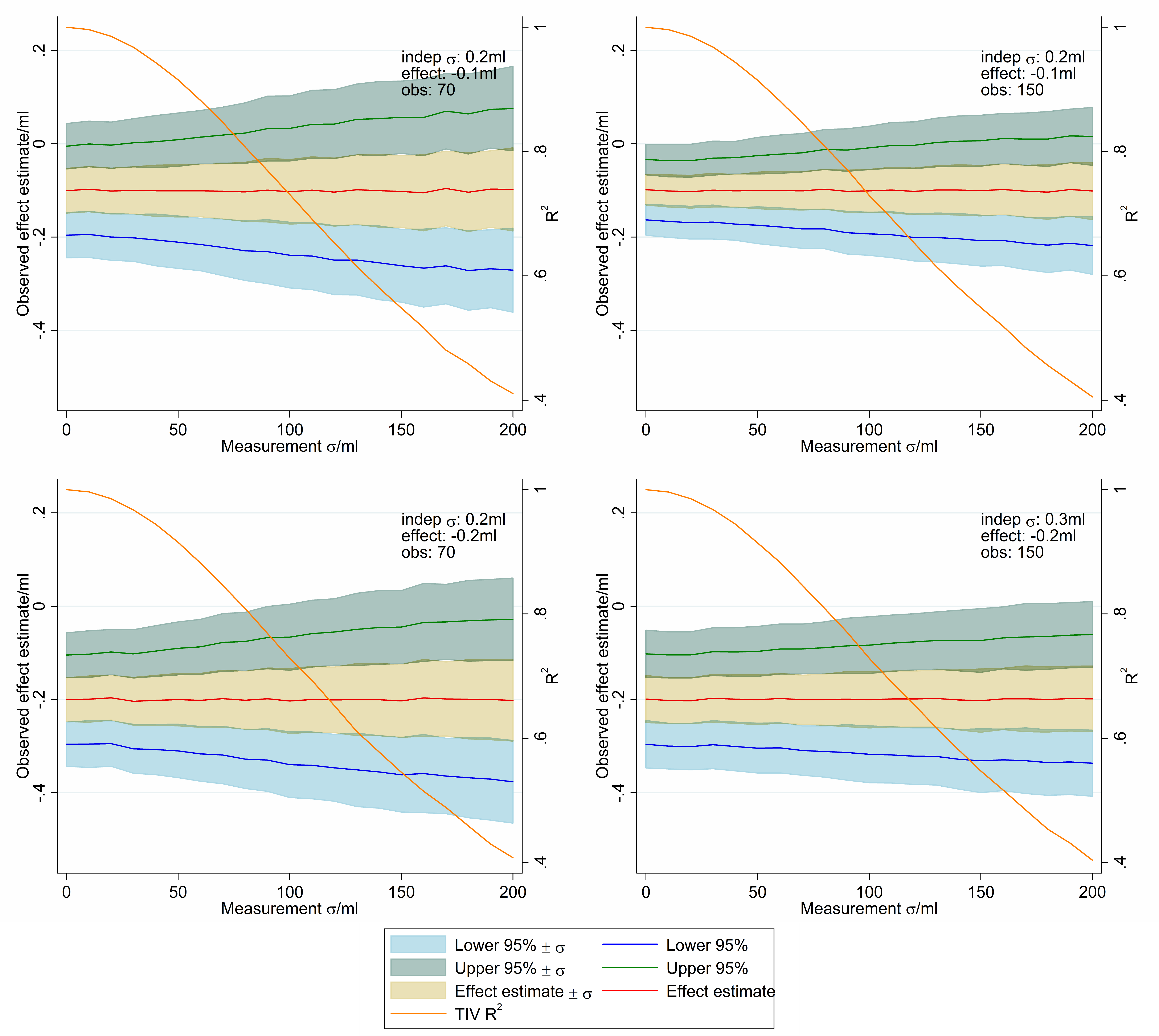
Supplementary Figure 2, effect estimates for a model of structural volume dependent on TIV. True effect size *effect*, additional structure variation *indep σ* (overall σ=0.44ml when *indep σ*=0.2ml), simulated study size *obs*. Each graph reflects the effect size and confidence intervals that would be observed under particular conditions with a certain error in TIV measurement (given the model below). The coloured areas reflect the standard deviation in each value (measured effect and confidence intervals) across experiments, which are not independent.

1. A normal distribution of TIV volumes was generated in Stata 12 with mean 1448ml and sd 165ml (reflecting the values from Nordenskjöld (2013))
2. “Measured” TIV values were generated by adding a normally distributed error with varying levels of standard deviation (Measurement σ).
3. Substructure volumes were generated by multiplying the “true” TIV values by 2.376x10^-3^ to produce a mean of 3.44ml (similar to the hippocampal values of Nordenskjöld) and a normal distribution added to these values (*indep σ*) which represents factors including inter-subject variation independent of TIV and measurement error.
4. The simulated observations were divided into two equal groups and a category effect added to the substructure volumes of one group.
5. Substructure volumes were regressed against group and “measured” TIV, and the estimated effect size and 95% confidence intervals found. This was repeated using Stata's simulate command for 1000 iterations at each data point and the standard deviation of estimated effect sizes and confidence intervals found. The mean R^2^ correlation of “measured” against “true” TIV was also calculated and is plotted against the right hand axis.

This is only an illustrative example and the impact of TIV measurement error will depend on many factors including sample size, relative degrees of variation dependent on and independent of TIV, the size of the effect of interest and distribution functions of errors and population volumes.
